# Residency in white-eared hummingbirds (*Hylocharis leucotis*) and its effect in territorial contest resolution

**DOI:** 10.7717/peerj.2588

**Published:** 2016-10-19

**Authors:** Verónica Mendiola-Islas, Carlos Lara, Pablo Corcuera, Pedro Luis Valverde

**Affiliations:** 1 Doctorado en Ciencias Biológicas y de la Salud, Universidad Autónoma Metropolitana-Iztapalapa, México, D.F., México; 2 Centro de Investigaciones en Ciencias Biológicas, Universidad Autónoma de Tlaxcala, Tlaxcala, Tlaxcala, Mexico; 3 Departamento de Biología, Universidad Autónoma Metropolitana-Iztapalapa, Mexico, D.F., México

**Keywords:** *Hylocharis leucotis*, Hummingbirds, Non-correlated asymmetry, Contest asymmetries, Prior residency, Territoriality

## Abstract

**Background:**

Territory owners usually defeat intruders. One explanation for this observation is the uncorrelated asymmetry hypothesis which argues that contests might be settled by an arbitrary convention such as “owners win.” We studied the effect of territorial residency on contest asymmetries in the white-eared hummingbird (*Hylocharis leucotis*) in a fir forest from central Mexico.

**Methods:**

Twenty white-eared male adult hummingbird territories were monitored during a winter season, recording the territorial behavior of the resident against intruding hummingbirds. The size and quality of the territory were related to the probability that the resident would allow the use of flowers by the intruder. Various generalized models (logistical models) were generated to describe the probabilities of victory for each individual resident depending on the different combinations of three predictor variables (territory size, territory quality, and intruder identity).

**Results:**

In general, small and low quality territory owners tend to prevent conspecific intruders from foraging at a higher rate, while they frequently fail to exclude heterospecific intruders such as the magnificent hummingbird (*Eugenes fulgens*) or the green violetear hummingbird (*Colibri thalassinus*) on any territory size. Our results showed that the identity of the intruder and the size and quality of the territory determined the result of the contests, but not the intensity of defense.

**Discussion:**

Initially, the rule that “the resident always wins” was supported, since no resident was expelled from its territory during the study. Nevertheless, the resident-intruder asymmetries during the course of a day depended on different factors, such as the size and quality of the territory and, mainly, the identity of the intruders. Our results showed that flexibility observed in contest tactics suggests that these tactics are not fixed but are socially plastic instead and they can be adjusted to specific circumstances.

## Introduction

Individuals who arrive first and establish ownership status in a given area containing resources seem to have a competitive advantage known as “prior-residence” (Kokko, López‐Sepulcre & Morrell, 2006). This phenomenon may be the result of an “uncorrelated assymetry” (Smith & Parker, 1976; Smith, 1982), such that all (or the majority) of the individuals in a population follow the rule “residents win” (i.e. Krebs, 1982; Tobias, 1997; Kemp & Wiklund, 2004). Although this rule prevents potentially harmful fights, alternative explanations suggest that its stability depends on asymmetries in fighting ability or resource value. For example, the resource-holding power hypothesis predicts that residents win because they possess traits that increase fighting ability such as large body size, weaponry, strength or aggressiveness (Smith & Parker, 1976). Alternatively, residents often win contests regardless of their fighting ability, which might be equal to or even lower than that of the intruder (Johnsson, Carlsson & Sundström, 2000). This might be due to an increased motivation to fight driven by a better knowledge of the disputed resource value (Leimar & Enquist, 1984; Enquist & Leimar, 1987; Hack, Thompson & Fernandes, 1997; Humphries et al., 2006).

Under these alternative scenarios, residents seem likely to invest more resources defending a high-quality territory. However, an increase in territory value can cause an increased intrusion rate (i.e. intruders with high fighting ability), which in turn causes an increased owner defending costs (Johnsson, Carlsson & Sundström, 2000). If the cost of defending the territory exceeds its value, the resident must eventually leave (Smith, 1982). Thus, asymmetries in resident-intruder traits related to fighting ability (i.e. body size) and territory value can affect both the structure and outcome of resident-intruder conflicts.

Hummingbirds (Trochilidae) have been a model system for the study of territorial behavior because of their specialized nectarivorous habit, their small territories and their high energetic demands, in addition to the feasibility of quantifying and manipulating their food sources (Dearborn, 1998). To defend their feeding territory, they use perches to survey their territory and agonistic behaviors such as chasing intruders (Powers & McKee, 1994). Most studies to date have used asymmetries in body size and quality of resources to explain territorial dominance in hummingbirds (Stiles & Wolf, 1970; Kodric-Brown & Brown, 1978; Ewald & Rohwer, 1980; Ewald, 1985; Powers, 1987; Tiebout, 1993; Powers & McKee, 1994; Dearborn & Wiley, 1993; Camfield, 2006). On the other hand, the assessment of uncorrelated asymmetries in the strict sense is poorly understood in this bird group. Moreover, the only previously related study confirmed that male adults of *Calypte anna* and *Archilochus alexandri* always win their territory contests against juveniles. According to the authors, this pattern could be explained by differences in flight energetic costs; the intruding juveniles have a greater cost than adults (Ewald & Rohwer, 1980). However, to our knowledge information about the possible role of residence status against territory intruders has not been incorporated into the study of hummingbirds.

In this study, we investigated the effect of territory residence in contest asymmetries in natural conditions using the white-eared hummingbird (*Hylocharis leucotis*) as a model. To achieve this goal, we followed the established territories of males and analyzed the role of prior-residence in the result of territory conflict against conspecific and heterospecific intruders. Specifically, we tested if asymmetries in body size among contenders and territory value would affect the outcome of resident-intruder conflicts. Thus, we predicted that larger birds (residents or intruders) will tend to win, and if this difference is large enough among contenders, it could outweigh ownership asymmetry. However, since the territory value is generally variable (in covered area and number of open flowers), it is possible to predict a differential investment in the owner’s defense, depending not only on the intruder body size but also on the territory quality that matches the costs of its defense. Thus, we predicted that a reduction in the territory size and in the number of flowers (the smallest economical size), will favor exclusion of intruders similar in size to the resident (i.e. conspecifics), minimizing the cost of territory ownership (Dearborn, 1998). However, residents of large territories with a high number of open flowers will tend to invest more (by chasing and avoiding successful intrusions) in order to exclude larger body size intruders (i.e. heterospecific intruders).

## Methods

### Study area

The fieldwork was carried out from December 2014 to April 2015 in a fir forest located at the National Park El Chico (hereafter referred as NPEC), in the state of Hidalgo, Mexico (98°43′52″O, 20°12′26″N; 2,950 a 3,030 msnm; Comisión Nacional de Áreas Naturales Protegidas, 2005). The climate in the NPEC is temperate-sub humid with summer rains. The average yearly temperature is less than 12 °C and its annual yearly precipitation is 800 mm (Instituto Nacional de Estadística Geografía e Informática, 1998). The vegetation in the study area is a mosaic of a fir (*Abies religiosa*), pine forest (*Pinus patula*, *P. montezumae*, *P. teocote* and *P. hartwegii*), and second-growth vegetation.

Our study focused on three of the seven hummingbird species recorded in the NPEC (Ortiz-Pulido & Vargas-Licona, 2008), the white-eared hummingbird (*H. leucotis*), the magnificent hummingbird (*Eugenes fulgens)*, and the green violet-ear (*Colibri thalassinus*), resident species in the study area. Throughout the winter season white-eared hummingbirds (*n* = 20 males, mean ± SE = 3.53 ± 0.75 g; *n* = 20 females, mean ± SE = 3.12 ± 0.21 g) mostly behave as territorial individuals, while magnificent (*n* = 20 males, mean ± SE = 7.25 ± 0.25 g; *n* = 20 females, mean ± SE = 7.03 ± 0.15 g) and green violet ear hummingbirds (*n* = 20 sex indistinguishable individuals, mean ± SE = 4.69 ± 0.34 g) behave rather as trapliners. Hummingbird reproductive activity has not previously been reported in the study area during the winter season (Márquez-Luna, Lara & Ortiz-Pulido, 2014), and although female white-eared hummingbirds may occasionally establish territories in this period, throughout the study the only territories found were male.

### Marking of hummingbirds

To recognize the identity of the residents and facilitate the recording of their territorial behaviors, we captured and marked individuals as soon as the winter flowering season began. For captures, we used 4–5 mist nets that were 6 and 12 m long which remained open from 09:00–14:00 h during two days in the flowering patches where the focal observations were being made. The captured individuals were marked with plastic markers made of melted colored beads (Perler beads, Wilton Brands Inc., Woodridge, IL, USA). These markers were then adhered to the back of the individuals with a quick-drying, non-toxic glue (Kola Loka, E. I. du Pont de Nemours and Company, Edo. de México). This marking technique is not harmful to hummingbirds and is known to be an efficient alternative for the visual identification of individuals in the field. Furthermore, it allows individual monitoring during several months without affecting their flight or behavior (Kapoor, 2012). Caught individuals were identified, sexed, weighed, and measured.

Marked individuals were observed defending feeding territories during various days after they were marked. None of these birds were found dead or missing during the study. Each marked individual had a color combination exclusively for its posterior identification. Thus, we were able to locate, identify, and record the behaviors of territorial males. The field research reported here was carried out with minimal bird manipulation using the required permits (SEMARNAT No. FAUT-0296) and followed the Guidelines for the Use of Wild Birds in Research by the North American Ornithological Council.

### Intruder-resident contest

After completing the marking process of the individuals, we were able to identify and monitor 20 territories belonging to adult male white-eared hummingbirds (*Hylocharis leucotis*). Resident status in a territory was considered if the resident hummingbird foraged inside of it, and defended it against intruders for several consecutive days, without spending time away from it even after chases. The resident hummingbird behavior was observed and recorded from 09:00 to 13:00 h during two consecutive days in each of the identified territories. The observation period was eight hours for each territory. Due to the size and the vegetation density of the territories, the observations were done from different points, at a distance of approximately 10 meters from it. This did not modify the behavior of the hummingbird. In each observation period, we recorded the number and species of intruders and the frequency of chases against them. Because resident hummingbirds always returned to their perches and foraged inside their territory, If intruding hummingbirds foraged inside the territory (i.e. introducing the bill into a flower), it was stated as a “successful intrusion;” however, the victory for a resident would only be stated based on its ability to expel an intruder from the territory without allowing it to feed.

### Territory quality and size

The number of flowers in each of the 20 territories was counted around the time that each owner was observed. In addition, we chose 20–30 flowers in the same condition of the plant species inside each territory to measure the nectar volume in a non-destructive way by using calibrated microcapillars (standing crop). Sugar concentration (sucrose percentage) was measured with a pocket refractometer (ATAGO, Master Refractometer 50 H, range concentration 0°–50° BRIX scale) then, the volume and concentration of nectar were used to quantify the variability of energy within each territory (kJ/flower). Both the volume (*n* = 20; *r* = 0.82, *p* < 0.05) and concentration (*n* = 20; *r* = 0.76, *p* < 0.05) were highly correlated with the number of flowers in a territory. Thus, this variable was used to determine whether the resident status was affected by the quality of the defended patch.

To establish the area (size) of each territory, we first observed the behavior of the resident hummingbird and the sites where the perch, foraging, and defensive activity (chases) took place; then, we established the coordinates “x” and “y” of the most external sites where the recorded activity by the resident of the territory took place. We calculated the area of the territory by using the minimum convex polygon (MCP) method with the software Biota™ 2.0 Alpha (Ecological Software Solutions LLC, 2004).

### Data analysis

Since there can be more than one predictor affecting the probability that a resident wins contests against the intruders, we generated various generalized models (logistic models involving a logit link and binomial error distribution) describing the probabilities of victory for each recorded individual resident. We adjusted the models with different combinations of the predictor variables (intruder type and two continuous variables included as covariates: territory size and territory quality). The model with the lowest Akaike information criterion (AIC) was selected as the best model (Burnham & Anderson, 2002); then, with the final model, we calculated the territorial resident probability of winning a contest against a conspecific or heterospecific intruder. These statistical analyses were performed using R (R Development Core Team, 2008).

## Results

### Characteristics of the territories

The average area and number of flowers within the territories were 374.6 ± 44 m^2^ (range from 103 to 853 m^2^) and 1,649.3 ± 73.79 flowers (range from 360 to 4,293 flowers), respectively. By the end of the winter flowering period, the vast majority of established territories (99%) consisted of *Salvia elegans* (*n* = 400 flowers; mean ± SE: nectar volume = 1.59 ± 0.29 μl; sugar concentration = 16.64 ± 1.29 BRIX; energy = 0.092 kJ/flower), and some individual plants of *Lonicera mexicana* (*n* = 180 flowers; mean ± SE: nectar volume = 0.80 ± 0.22 μl; sugar concentration = 12.88 ± 1.70 BRIX; energy = 0.039 kJ/flower), *Senecio angulifolius* (*n* = 40 flowers; mean ± SE: nectar volume = 0.21 ± 0.11 μl; sugar concentration = 7.19 ± 0.45 BRIX; energy = 0.0044 kJ/flower/day), and *Cestrum roseum* (*n* = 40 flowers; mean ± SE: nectar volume = 0.94 ± 0.44 μl; sugar concentration = 2.56 ± 0.06 BRIX; energy = 0.004 kJ/flower). Thus, territories containing more flowers represented a higher energy content.

### Resident-intruder asymmetries

We registered the behavior of the territory residents over 160 h, obtaining 197 intrusive events of which, 110 were conspecific (males and females), and 87 were heterospecific (*Eugenes fulgens* and *Colibri thalassinus*). Of these we recorded 55 chase events with conspecific intruders and 32 heterospecific ones. In the event of an owner not chasing an intruder, it would remain perched emitting vocalizations. Although each territory was evaluated only during two consecutive days, some of the studied owners remained defending their feeding territories for several weeks. None of the resident hummingbirds followed during the study were expelled from the defended territory. Even though some individuals foraged inside the established territories during intraspecific interactions, intruders were eventually expelled. On the other hand, in interspecific interactions against individuals of *E. fulgens* and *C. thalassinus*, the intruder usually used the territory of the residents as perch and feeding sites. When the resident hummingbird tried to chase them, they would remain foraging, perching, and/or the chases would become prolonged.

### Effects of territory size, territory quality, and intruder type

We generated models with interactions between factors to evaluate if the size (area), quality (number of flowers), and intruder type affected the probability that the territorial resident would win the contests by chasing intruders or avoiding successful intrusions. The obtained models showed that intruder type is the best predictor of whether an intruder is chased or not (Table 1), while the evaluated characteristics of the defended territory had no significant effect (Table 1). Residents were more likely to chase conspecific than heterospecific intruders. Thus, asymmetries in body size among contenders, but not territory value, affected the probability of an owner chasing an intruder.

**Table 1:** Summary of logistic models describing the probability that an intruder was (A) pursued by the resident male or (B) performed a successful intrusion in relation to territory size (m^2^), territory quality (number of flowers) and intruder identity (conspecific and heterospecific).

(A) Chases						
Effect	AIC	Δ_*i*_	*df*	*z*	*p*	Intercept
Intruder type	269.97	0	196	1.84	0.0628	−2.436
Territory size × intruder type	270.95	0.98	196	−1.534	0.1251	1.439
Territory quality	272.48	2.51	196	1.185	0.2362	−1.866
Territory quality × intruder type	273.00	3.03	196	1.015	0.310	−1.531
Territory size	274.38	4.41	196	−0.169	0.866	−0.500
Territory size × territory quality × intruder type	274.66	4.69	196	0.719	0.472	0.944
Territory size × territory quality	276.41	6.44	196	0.741	0.458	0.184

(B) Successful intrusions						
Effect	AIC	Δ_*i*_	*df*	*z*	*p*	Intercept
Territory size × intruder type	223.69	0	196	−2.41	0.0001	1.092
Territory quality × intruder type	231.76	8.07	196	−1.32	0.0184	−1.313
Intruder type	234.66	10.97	196	−4.67	0.0002	0.322
Territory size × territory quality	239.07	15.38	196	−2.88	0.0039	−3.581
Territory size	246.26	22.57	196	−0.605	0.5451	−1.266
Territory quality	254.88	31.19	196	2.960	0.1844	−4.796

**Notes:**

Models ranked in increasing order of AIC values.

Δ_*i*_ represents the difference between the AIC value of model and the AIC value of the most parsimonious model.

**Figure 1:**
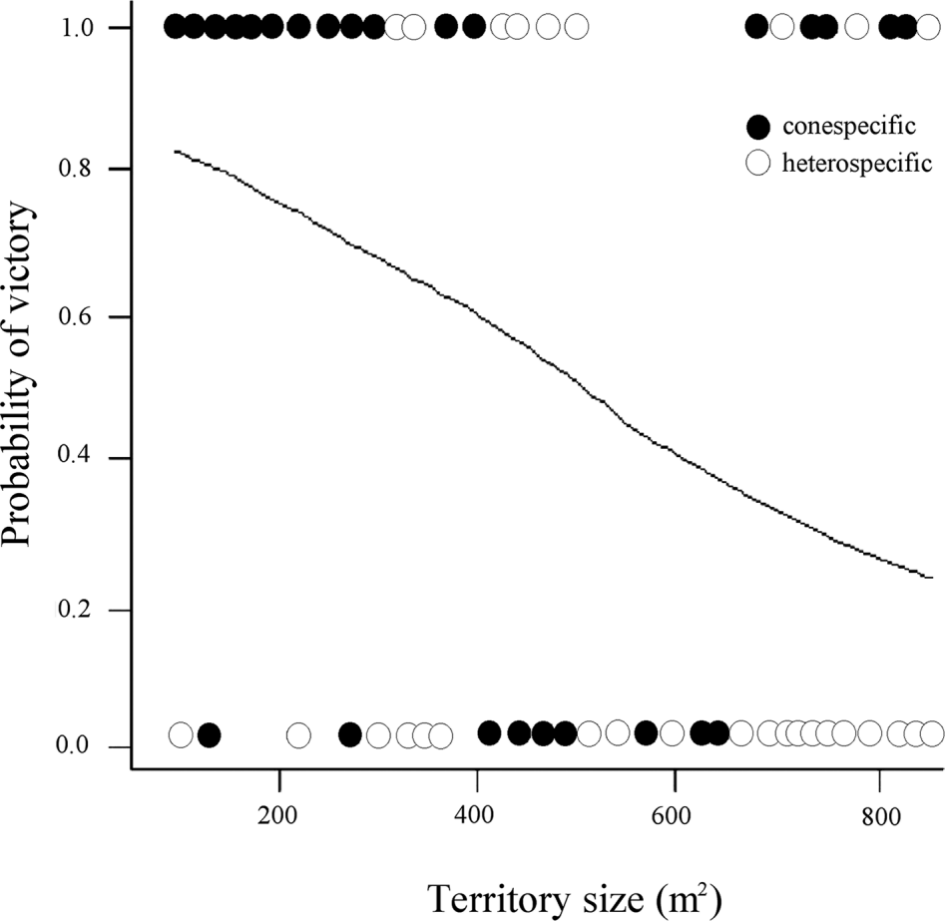
Probability of victory of resident male white-eared hummingbirds in relationship to the size (area) of the territory and the identity of the intruders. Each circle illustrates whether a conspecific or heterospecific intruder was expelled from the territory without (0) or allowing (1) it to feed (successful intrusion). The fitted line reflects the modeled probability of intruders visiting territories covering different areas (m^2^), showing that compared with heterospecific intruders, conspecific intruders had a higher probability to be expelled from small territories without being able to feed.

On the contrary, the model including territory size interacting with intruder type showed strong support to explain whether a resident avoided or not successful intrusions (Table 1; Fig. 1). Three more models with lower AIC values included the effect of intruder type, and the interaction between territory size and intruder type, and the interaction between size and quality of territory (Table 1). Overall, the residents have a higher probability of victory (avoiding intruder use of their nectar resources) against conspecific intruders in territories of smaller size and lower quality, while in the interspecific interactions, their probability of victory was less in all cases.

## Discussion

The male white-eared hummingbirds (*H. leucotis*) seem to use different criteria to determine victory in contests against intruders. In general terms, the rule of “the resident always wins” seemed to be true, since no resident was expelled from its feeding territory during the study. However, the resident-intruder asymmetries were important when considering the size and quality of defended resources, as well as the identity of the intruders (mainly when there are differences in size or weight). These aspects are the main factors to determine the result of the contests during the territorial defense of these hummingbird species. Even though residents tended to show agonistic behaviors (such as the chasing) regardless of the type and size of the intruder and the quality of the defended territory, their tolerance for allowing the use of the defended flowers was differential.

The only species that establishes and defends feeding territories during long periods of time (weeks) through winter in the NPEC is the white-eared hummingbird. The magnificent hummingbird and the green violetear hummingbird behave mainly as trapliners (V. Mendiola-Islas, 2016, personal observation). Apparently, their size gives them an advantage when the resident hummingbirds are trying to expel them. It has been suggested that body size is an important factor of dominance systems in hummingbirds (Dearborn, 1998). The larger-sized species have higher energetic requirements, reflected in typical movement patterns to look for resources within different habitats (marauder or trapliner) (Des Granges, 1978). On the other hand, smaller hummingbirds generally are incapable of chasing the larger ones and are forced to obtain their food by non-territorial foraging (del Coro Arizmendi & Ornelas, 1990). However, in our study we found that in spite of their size (the smallest of the three species in the study site), the white-eared hummingbird establishes and defends food territories, from both conspecific and heterospecific individuals. The identity of the intruder was not enough to expel the owners from their territories. When the individuals of the larger-sized species entered a territory, the resident would expel them by chasing and vocalizing during a few seconds until the larger species would finally take over and use the flowers inside the territory. On the other hand, in the event of intruders belonging to the same species, they would feed successfully a few times, but then be chased and expelled without having confronted the resident.

We found that small territory owners (covered area in m^2^) tend to prevent conspecific intruders from foraging at a higher rate, while they frequently fail to exclude heterospecific intruders on any territory size. Thus, our results are consistent with previous studies on territory economy predicting that hummingbirds appeared to be defending territories of the smallest economical size to minimize the cost of territory ownership and to maximize the time spent sitting (Hixon, Carpenter & Paton, 1983; Powers, 1987; Dearborn, 1998; Cotton, 1998). Differences in the success rate of defense against intruders of different body sizes can be explained by (1) the size asymmetries among contenders and (2) detectability of intruders in relation to the size of the territory. It has been suggested that intruders should be chased/excluded based on the potential cost associated with engaging them in aggressive contests (Krebs & Davies, 1993). Thus, intruders’ exclusion frequency should have an inverse relationship with their body size (Dearborn, 1998). This prediction is supported by our results because larger sized intruders (heterospecifics) had a lower rate of exclusion than similar sized intruders (conspecifics) regardless of the territory quality. Interestingly, the outcome of disputes among similarly sized contenders would vary as the territory size increased. Small intruders should be more difficult for the territory owner to detect (Feinsinger & Colwell, 1978). However, the smallest ones were more likely to be excluded from smaller rather than from larger territories. This result suggests that resident birds were less able to detect conspecific intruders as territory size increased.

As previously mentioned, we can suggest that the response of the resident white-eared hummingbird to the intruder highly depends on the difference in body size. However, there are several examples in hummingbirds where depending on life history stage (breeding/non-breeding; pre-migratory stages), hormone levels, feeding mode (territorial or trapliners), sex, and abiotic factors (i.e. Stiles & Wolf, 1970; Kodric-Brown & Brown, 1978; Ewald, 1985; Cotton, 1998; Sandlin, 2000; González-Gómez et al., 2014), the body size among species (heterospecifics) can be irrelevant as a variable to explain the results of contests over territory ownership. Conversely, at the intra-specific level (conspecifics), within the same sex, body size can be a very important factor in the result of territorial fights (Carpenter et al., 1993). The relationship between body size and the probability of winning agonistic confrontations has been observed in diverse hummingbird communities (Feinsinger, 1976; Lyon, 1976; Feinsinger & Colwell, 1978; del Coro Arizmendi & Ornelas, 1990; Lara, 2006). Recently, it has been hypothesized that smaller species have a greater probability of being dominant in aggressive contexts when interacting with species that are evolutionarily distantly related (Martin & Ghalambor, 2014). The white-eared hummingbird belongs to a different (“Emeralds”) clade than *E. fulgens* (“Mountain Gems”) and *C. thalassinus* (“Mangos”). It is also distantly related to these species. This hypothetical prediction may partially explain why larger species did not expel the white-eared hummingbirds from the territories they defended. In an evolutionary context, these interactions still require additional research.

Territorial residence plays an important role in contested asymmetries in the studied systems, as has been shown both in the laboratory and natural conditions (Krebs, 1982; Figler & Einhorn, 1983; Beletsky & Orians, 1989; Dearborn & Wiley, 1993; Tobias, 1997; Umbers, Osborne & Keogh, 2012). We have shown that the advantage of residence in the white-eared hummingbird depends on the asymmetries in the size of defended resources and the size of the opponents. As it has been mentioned, the particular dependence on floral nectar as a primary food source, combined with the characteristic distribution pattern of the plants that provide it, causes territoriality in hummingbirds to be a fundamental structuring force of their communities. Our study provides additional information about a mechanism not previously evaluated in the Trochilidae, which deserves to be analyzed under controlled conditions.

None of the three principal hypothesizes formulated to explain the result of the contests can explain the territorial behavior by themselves. Asymmetries vary depending on age, sex, and mating system of the different bird species. The flexibility observed in contest tactics suggests that, even though the first models provide useful information, these tactics are not fixed but are socially plastic instead and they can be adjusted to specific circumstances. In further studies, it is necessary to consider the relationship between all of the asymmetries in order to understand under what circumstances territorial dominance occurs.

## Supplemental Information

10.7717/peerj.2588/supp-1 Supplemental Information 1 The raw data on the characteristics of the territories owned by white-eared hummingbird males and their territorial behavior against intruders applied for data analysis and preparation for Fig. 1.

## Additional Information and Declarations

### Field Study Permissions

The following information was supplied relating to field study approvals (i.e., approving body and any reference numbers): The field research reported here was performed using the required permit (SEMARNAT No. FAUT-0296).

### Data Deposition

The following information was supplied regarding data availability: The raw data has been supplied as Supplemental Dataset Files.
