# Supplementary material for: Residency in white-eared hummingbirds (Hylocharis leucotis) and its effect in territorial contest resolution
Source: PeerJ. 2016 Oct 19;4:e2588. doi: 10.7717/peerj.2588 (PMC5075688; doi:10.7717/peerj.2588)
Supplement: Supplemental Information 1 [file peerj-04-2588-s001.docx]

Suplemental data. Characteristic of the territories ownered by male White-eared hummingbirds and followed through the study, showing their records of succesful (1) or failed (0) chases and intrusions against conspecific (cons) and heterospecific (hetero) intruders.

| Territory | Covered área (m^2^) | Size category | Number of open flowers | Quality category | Chases | Intruder type | Intrusions |
| --- | --- | --- | --- | --- | --- | --- | --- |
| 1 | 607.48 | large | 514 | poor | 0 | cons | 1 |
|  |  |  |  |  | 0 | cons | 0 |
|  |  |  |  |  | 0 | cons | 0 |
|  |  |  |  |  | 1 | cons | 1 |
|  |  |  |  |  | 0 | cons | 0 |
|  |  |  |  |  | 0 | cons | 0 |
|  |  |  |  |  | 0 | cons | 0 |
|  |  |  |  |  | 0 | cons | 1 |
|  |  |  |  |  | 0 | cons | 1 |
| 2 | 344.37 | small | 867 | poor | 0 | cons | 0 |
|  |  |  |  |  | 1 | cons | 0 |
|  |  |  |  |  | 0 | cons | 0 |
|  |  |  |  |  | 0 | cons | 1 |
|  |  |  |  |  | 0 | cons | 0 |
|  |  |  |  |  | 1 | cons | 0 |
|  |  |  |  |  | 1 | cons | 1 |
|  |  |  |  |  | 1 | cons | 0 |
|  |  |  |  |  | 1 | cons | 0 |
|  |  |  |  |  | 0 | cons | 1 |
|  |  |  |  |  | 0 | cons | 0 |
|  |  |  |  |  | 0 | cons | 0 |
|  |  |  |  |  | 0 | cons | 0 |
|  |  |  |  |  | 0 | cons | 1 |
|  |  |  |  |  | 0 | cons | 0 |
|  |  |  |  |  | 1 | cons | 0 |
| 3 | 552.885 | large | 1037 | poor | 0 | cons | 0 |
|  |  |  |  |  | 1 | cons | 1 |
|  |  |  |  |  | 1 | cons | 0 |
|  |  |  |  |  | 0 | cons | 0 |
|  |  |  |  |  | 0 | cons | 1 |
|  |  |  |  |  | 0 | cons | 0 |
|  |  |  |  |  | 0 | cons | 0 |
|  |  |  |  |  | 0 | cons | 1 |
|  |  |  |  |  | 0 | cons | 0 |
|  |  |  |  |  | 0 | cons | 0 |
|  |  |  |  |  | 0 | cons | 0 |
|  |  |  |  |  | 0 | cons | 1 |
|  |  |  |  |  | 1 | cons | 0 |
|  |  |  |  |  | 1 | cons | 0 |
| 4 | 346.165 | small | 1562 | high | 0 | cons | 0 |
|  |  |  |  |  | 1 | cons | 1 |
|  |  |  |  |  | 0 | cons | 0 |
|  |  |  |  |  | 1 | cons | 0 |
|  |  |  |  |  | 0 | cons | 0 |
|  |  |  |  |  | 1 | cons | 0 |
|  |  |  |  |  | 1 | cons | 1 |
|  |  |  |  |  | 1 | cons | 0 |
|  |  |  |  |  | 1 | cons | 0 |
|  |  |  |  |  | 1 | cons | 1 |
|  |  |  |  |  | 0 | cons | 1 |
|  |  |  |  |  | 1 | cons | 1 |
|  |  |  |  |  | 0 | cons | 1 |
| 5 | 283.89 | small | 1249 | high | 0 | cons | 1 |
|  |  |  |  |  | 1 | cons | 0 |
|  |  |  |  |  | 1 | cons | 1 |
|  |  |  |  |  | 1 | cons | 0 |
|  |  |  |  |  | 0 | cons | 1 |
|  |  |  |  |  | 1 | cons | 1 |
|  |  |  |  |  | 0 | cons | 1 |
|  |  |  |  |  | 0 | cons | 0 |
| 6 | 503.03 | large | 4293 | high | 0 | cons | 1 |
|  |  |  |  |  | 0 | cons | 1 |
|  |  |  |  |  | 1 | cons | 0 |
|  |  |  |  |  | 0 | cons | 1 |
|  |  |  |  |  | 0 | cons | 1 |
|  |  |  |  |  | 0 | cons | 1 |
|  |  |  |  |  | 0 | cons | 1 |
|  |  |  |  |  | 1 | cons | 0 |
|  |  |  |  |  | 1 | hetero | 0 |
|  |  |  |  |  | 1 | hetero | 0 |
|  |  |  |  |  | 0 | hetero | 0 |
|  |  |  |  |  | 1 | hetero | 0 |
|  |  |  |  |  | 1 | hetero | 1 |
|  |  |  |  |  | 1 | hetero | 0 |
|  |  |  |  |  | 1 | hetero | 0 |
|  |  |  |  |  | 0 | hetero | 0 |
| 7 | 429.61 | large | 2426 | high | 0 | hetero | 0 |
|  |  |  |  |  | 0 | hetero | 1 |
|  |  |  |  |  | 1 | hetero | 0 |
|  |  |  |  |  | 0 | hetero | 1 |
|  |  |  |  |  | 1 | hetero | 0 |
|  |  |  |  |  | 0 | hetero | 1 |
|  |  |  |  |  | 1 | hetero | 0 |
|  |  |  |  |  | 1 | hetero | 0 |
|  |  |  |  |  | 1 | hetero | 0 |
|  |  |  |  |  | 1 | hetero | 0 |
|  |  |  |  |  | 0 | hetero | 1 |
|  |  |  |  |  | 1 | hetero | 1 |
|  |  |  |  |  | 0 | hetero | 0 |
|  |  |  |  |  | 0 | hetero | 0 |
|  |  |  |  |  | 1 | hetero | 0 |
|  |  |  |  |  | 0 | hetero | 0 |
|  |  |  |  |  | 0 | hetero | 1 |
|  |  |  |  |  | 0 | hetero | 1 |
|  |  |  |  |  | 0 | hetero | 0 |
|  |  |  |  |  | 1 | hetero | 0 |
|  |  |  |  |  | 1 | hetero | 1 |
|  |  |  |  |  | 1 | hetero | 0 |
|  |  |  |  |  | 1 | hetero | 0 |
|  |  |  |  |  | 1 | hetero | 1 |
|  |  |  |  |  | 1 | hetero | 0 |
| 8 | 409.21 | large | 1387 | high | 0 | hetero | 0 |
|  |  |  |  |  | 1 | hetero | 1 |
|  |  |  |  |  | 1 | hetero | 0 |
|  |  |  |  |  | 1 | hetero | 1 |
|  |  |  |  |  | 1 | hetero | 0 |
|  |  |  |  |  | 0 | hetero | 1 |
|  |  |  |  |  | 0 | hetero | 0 |
|  |  |  |  |  | 0 | hetero | 0 |
|  |  |  |  |  | 0 | hetero | 1 |
|  |  |  |  |  | 1 | hetero | 0 |
|  |  |  |  |  | 0 | cons | 1 |
|  |  |  |  |  | 0 | cons | 1 |
|  |  |  |  |  | 0 | cons | 1 |
|  |  |  |  |  | 0 | cons | 0 |
|  |  |  |  |  | 0 | cons | 1 |
|  |  |  |  |  | 1 | cons | 1 |
|  |  |  |  |  | 1 | cons | 1 |
|  |  |  |  |  | 1 | cons | 1 |
| 9 | 306.95 | small | 3335 | high | 1 | cons | 0 |
|  |  |  |  |  | 0 | cons | 1 |
|  |  |  |  |  | 0 | cons | 1 |
|  |  |  |  |  | 0 | cons | 1 |
|  |  |  |  |  | 1 | cons | 1 |
|  |  |  |  |  | 1 | cons | 1 |
|  |  |  |  |  | 1 | cons | 1 |
|  |  |  |  |  | 0 | cons | 1 |
|  |  |  |  |  | 0 | cons | 1 |
|  |  |  |  |  | 0 | cons | 1 |
| 10 | 302.55 | small | 789 | poor | 0 | cons | 0 |
|  |  |  |  |  | 0 | hetero | 0 |
|  |  |  |  |  | 1 | hetero | 0 |
|  |  |  |  |  | 1 | hetero | 0 |
| 11 | 606.395 | large | 1580 | high | 1 | hetero | 0 |
|  |  |  |  |  | 0 | hetero | 0 |
|  |  |  |  |  | 0 | hetero | 0 |
| 12 | 228.35 | small | 1061 | poor | 0 | hetero | 0 |
|  |  |  |  |  | 0 | hetero | 0 |
|  |  |  |  |  | 1 | hetero | 0 |
|  |  |  |  |  | 1 | hetero | 0 |
| 13 | 142.85 | small | 1068 | poor | 1 | hetero | 0 |
|  |  |  |  |  | 1 | hetero | 0 |
|  |  |  |  |  | 0 | hetero | 1 |
|  |  |  |  |  | 0 | hetero | 0 |
|  |  |  |  |  | 0 | hetero | 0 |
|  |  |  |  |  | 1 | hetero | 0 |
|  |  |  |  |  | 0 | hetero | 0 |
|  |  |  |  |  | 0 | hetero | 0 |
|  |  |  |  |  | 0 | hetero | 0 |
|  |  |  |  |  | 0 | hetero | 0 |
|  |  |  |  |  | 0 | hetero | 0 |
|  |  |  |  |  | 0 | hetero | 1 |
|  |  |  |  |  | 1 | hetero | 0 |
|  |  |  |  |  | 0 | hetero | 1 |
| 14 | 103.28 | small | 1293 | high | 0 | hetero | 0 |
|  |  |  |  |  | 1 | hetero | 0 |
|  |  |  |  |  | 0 | hetero | 0 |
| 15 | 216.66 | small | 1408 | high | 0 | hetero | 0 |
|  |  |  |  |  | 1 | hetero | 0 |
|  |  |  |  |  | 0 | hetero | 0 |
|  |  |  |  |  | 0 | hetero | 0 |
|  |  |  |  |  | 1 | hetero | 0 |
|  |  |  |  |  | 0 | hetero | 0 |
| 16 | 282.6 | small | 869 | poor | 1 | hetero | 0 |
|  |  |  |  |  | 1 | hetero | 0 |
|  |  |  |  |  | 1 | hetero | 1 |
|  |  |  |  |  | 1 | hetero | 0 |
|  |  |  |  |  | 0 | hetero | 0 |
|  |  |  |  |  | 1 | hetero | 0 |
| 17 | 853.54 | large | 983 | poor | 1 | hetero | 1 |
|  |  |  |  |  | 0 | hetero | 0 |
|  |  |  |  |  | 0 | hetero | 0 |
|  |  |  |  |  | 0 | hetero | 0 |
|  |  |  |  |  | 0 | hetero | 0 |
|  |  |  |  |  | 1 | hetero | 0 |
|  |  |  |  |  | 0 | hetero | 0 |
|  |  |  |  |  | 0 | hetero | 0 |
|  |  |  |  |  | 0 | hetero | 0 |
|  |  |  |  |  | 1 | hetero | 0 |
|  |  |  |  |  | 1 | hetero | 1 |
|  |  |  |  |  | 1 | hetero | 0 |
|  |  |  |  |  | 1 | hetero | 0 |
| 18 | 222.48 | small | 360 | poor | 1 | hetero | 0 |
| 18 |  |  |  |  | 0 | hetero | 0 |
| 19 | 677.1 | large | 1283 | high | 1 | hetero | 0 |
| 19 |  |  |  |  | 0 | hetero | 0 |
| 19 |  |  |  |  | 1 | hetero | 0 |
| 19 |  |  |  |  | 1 | hetero | 0 |
| 19 |  |  |  |  | 0 | hetero | 0 |
| 19 |  |  |  |  | 1 | hetero | 0 |
| 19 |  |  |  |  | 0 | hetero | 1 |
| 19 |  |  |  |  | 1 | hetero | 0 |
| 19 |  |  |  |  | 0 | hetero | 0 |
| 19 |  |  |  |  | 0 | hetero | 0 |
| 19 |  |  |  |  | 0 | hetero | 0 |
| 19 |  |  |  |  | 1 | hetero | 0 |
| 20 | 533.91 | large | 418 | poor | 0 | hetero | 0 |
